# ALIGNING ASSESSMENTS AND MEASURES OF CAREGIVER NEGLECT WITH INTERVENTION DEVELOPMENT: NEW INSIGHTS AND DIRECTIONS

**DOI:** 10.1093/geroni/igad104.0450

**Published:** 2023-12-21

**Authors:** E-Shien Chang, David Hancock, Alan Stevens

## Abstract

As one of the most common forms of elder mistreatment, caregiver neglect is associated with significant morbidity and mortality. The unique stresses, demands, and challenges associated with family caregiving likely increases the risk of neglect. Addressing the multifaceted nature of caregiver neglect in diverse family caregivers of older persons necessitates alignment of meaningful assessments and methodologies with evidence-based intervention development. Gaps in knowledge that urgently need filling include refining conceptual and operational definitions of caregiver neglect, validating novel measurement approaches, and identifying effective caregiver-centered study engagement and recruitment strategies. To address these gaps, this symposium will draw upon insights from four caregiver neglect studies across community and clinical settings. Dr. David Hancock will characterize prevalence and correlates of caregiver neglect using unmet activities of daily living needs in a sample of dementia patients and their caregivers. Dr. E-Shien Chang will test the impact of varying operational definitions of caregiver neglect on prevalence estimates and associated risk and protective factors. Dr. Tony Rosen will present a novel, validated caregiver neglect severity measure to more accurately assess the impact of neglect on older persons’ health and quality of life outcomes. Dr. Farida Ejaz will highlight challenges and successes in identifying and involving family caregivers at risk of neglectful caregiving behaviors to inform intervention. Finally, Dr. Alan Stevens, as policy and advocacy expert for family caregivers, will provide summative comments and recommendations for policy and practice changes to better reduce risk of neglect and improve support for family caregivers.

